# A corpus of plant–disease relations in the biomedical domain

**DOI:** 10.1371/journal.pone.0221582

**Published:** 2019-08-28

**Authors:** Baeksoo Kim, Wonjun Choi, Hyunju Lee

**Affiliations:** School of Electrical Engineering and Computer Science, Gwangju Institute of Science and Technology, Buk-gu, Gwangju, South Korea; Politechnika Krakowska im Tadeusza Kosciuszki, POLAND

## Abstract

**Background:**

Many new medicines have been derived from natural sources such as plants, which have a long history of being used for disease treatment. Thus, their benefits and side effects have been studied, and plant-related information including plant and disease relations have been accumulated in Medline articles. Because numerous articles are available in Medline and are written in natural language, text-mining is important. However, a corpus of plant and disease relations is not available yet. Thus, we aimed to construct such a corpus.

**Methods and results:**

In this study, we designed and annotated a plant–disease relations corpus, and proposed a computational model to predict plant–disease relations using the corpus. We categorized plant and disease relations into four types: treatments of diseases, causes of diseases, associations, and negative relations. To construct a corpus of plant–disease relations, we first created its annotation guidelines and randomly selected 200 Medline abstracts. From these abstracts, we identified 1,405 and 1,755 plant and disease mentions, annotated to 105 and 237 unique plant and disease identifiers, respectively. When we selected sentences containing at least one plant and one disease mention, we extracted 878 plant and 1,077 disease entities, which finally generated a corpus of plant-disease relations including 1,309 relations from 199 abstracts. To verify the effectiveness of the corpus, we proposed a convolutional neural network model with the shortest dependency path (SDP-CNN) and applied it to the constructed corpus. The micro F-score with ten-fold cross-validation was found to be 0.764. We also applied the proposed SDP-CNN model to all Medline abstracts. When we measured its performance for 483 randomly selected plant-disease co-occurring sentences, the model showed a precision of 0.707.

**Conclusion:**

The plant–disease relations corpus is unique and represents an important resource for biomedical text-mining. The corpus of plant and disease relations is available at http://gcancer.org/pdr/.

## Introduction

Empirical knowledge about plant use for treating disease has increased over thousands of years [1, 2], and natural products including plants have become a starting point for successful drug development such as artemisinin for treating malaria [3]. Nonetheless, for many medicinal plants, the mechanisms of action underlying disease treatment have not been revealed yet. Because plants are composed of a variety of chemicals that act on a variety of targets, it is necessary to examine the action of a plant itself as well as the action of single chemicals [4]. Thus, the results of biomedical research, including relations between plants and diseases, have been reported in the Medline database. Although several text-mining studies have been conducted to identify information from Medline abstracts, there are few studies on plant–disease relations.

Several steps are required to extract structured information from unstructured Medline abstracts [5, 6]. We first have to define a format of the structured information to extract, such as the entity types and relation types. It is then necessary to automatically recognize target entity names and relations between the recognized entities using rule-based or machine learning techniques. Because supervised learning requires training and test data for learning and evaluating algorithms, respectively, construction of a corpus for training and test data is essential. To the best of our knowledge, research on the relations between plants and diseases has not been addressed systematically. Therefore, this study began with the definition of plant names, disease names, relations between plants and diseases, and then created a corpus for these defined relations.

Wan et al. (2016) compiled a corpus for the analysis of TCM literature. The corpus was constructed with five relation types: herb–syndrome, herb–disease, formula–syndrome, formula–disease, and syndrome–disease [7]. However, because that study targeted Chinese literature, it is impossible for this method to analyze articles published in English. Although a corpus for plant and disease relations constructed from Medline articles is not yet available, corpora for chemical and disease relations have been constructed. Li et al. (2015) annotated chemicals, diseases, and chemical-induced disease (CID) forming a corpus for the BioCreative V chemical–disease relation task. This corpus used chemical and disease information from existing Comparative Toxicogenomics Database (CTD)-Pfizer corpus [8] and CID annotation for 1,500 articles. Schlaf et al. (2013) created a corpus of the relations between chemicals and diseases for USPTO patent literature.

Few text-mining studies regarding plants and their medicinal effects have also been conducted. Wu et al. (2004) studied the relation between Traditional Chinese Medicine (TCM), symptoms, and genes in Medline abstracts; this study was one of the first to use text-mining to identify biomedical relations in TCM [9]. In that study, co-occurrences of terms were used to extract the relations between entities. TCMGeneDIT [10] is a database that includes rule-based information extracted for TCM–gene, TCM–disease, TCM–ingredient, TCM–effect, TCM–gene–disease, and gene–ingredient relations. However, the TCM associations (except for TCM effects) extracted by means of term co-occurrence and statistical methods are less reliable. In ThaiHerbMiner [11], the relations among traditional Thai medicine, genes, and diseases were extracted via co-occurrence of triplets with causal verbs. That study has an advantage of using causal verbs rather than simple co-occurrences. Nevertheless, if relations are described with words not included in the causal-verb list, they are not recognized.

In this study, we designed and constructed a corpus of plant and disease entities and their relations. To verify the usefulness and reliability of the constructed corpus, we propose a convolutional neural network with the shortest dependency paths (SDP-CNN) model and apply it to the constructed plant–disease corpus. This study is expected to be an important resource for research on relations between plants and diseases.

## Materials and methods

In this section, we first introduce the definition of plant and disease relations and the guidelines for constructing a corpus of plant–disease relations. Then, for corpus construction, we describe a procedure for selecting Medline abstracts and an annotation tool, followed by a subsection on the evaluation of corpus quality. Finally, a plant–disease relation prediction method is presented.

### A definition of plant and disease relations

**Fig 1:**
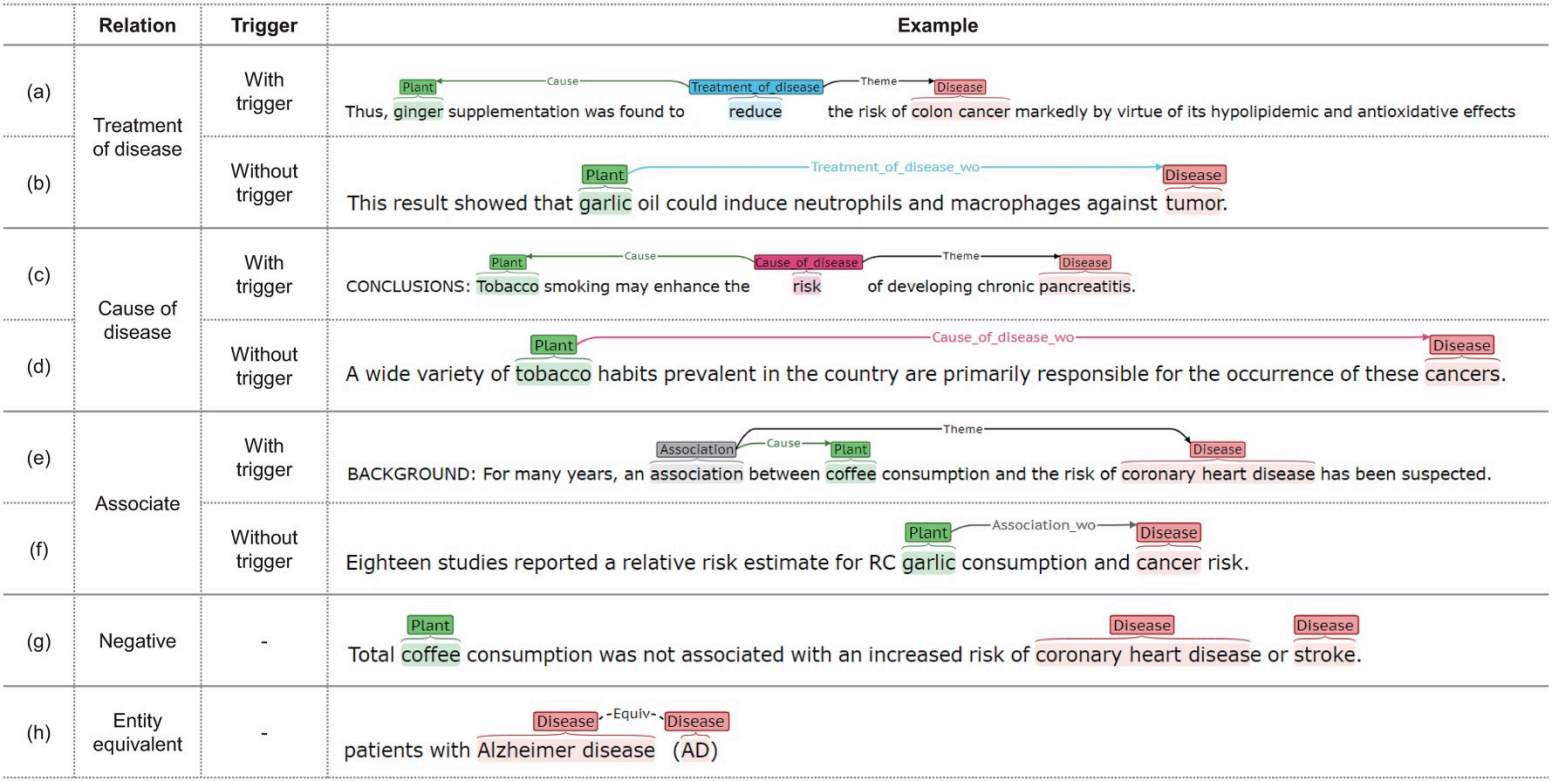
Examples of relations and their annotations. (a) A treatment of disease relation with a trigger (PMID:20021021). (b) A treatment of disease relation without a trigger (PMID: 20622705). (c) A cause of disease relation with a trigger (PMID: 20622705). (d) A cause of disease relation without a trigger (PMID: 2814139). (e) An association relation with a trigger (PMID:2215561). (f) An association relation without a trigger (PMID: 11010950). (g) A negative relation (PMID:2215561). (h) An equivalence relation (PMID: 9823823).

In this study, we aimed to annotate the relations between plants and diseases in Medline articles. Sentences showing a relation between a plant and disease can be categorized into four cases as shown in Fig 1. Relations of plants ingested for the treatment or alleviation of diseases involve (plant)–(treat)–(disease) descriptions. In this study, the relations between these are defined by *treatment of disease (ToD)* relations (Fig 1(a) and 1(b)). For relations of ingested plants with causes of diseases, there is a (plant)-(cause)-(disease) description. These relations are defined by *cause of disease (CoD)* relations (Fig 1(c) and 1(d)). Sentences in which it is difficult to distinguish between ToD and CoD, even though they show relations between plants and diseases, are annotated as an *association* relation (Fig 1(e) and 1(f)).

In a sentence containing plant and disease names, a relation can be expressed with or without explicit words or phrases describing the relation. These explicit words or phrases are called triggers. Fig 1(a), 1(c) and 1(e) show relations involving triggers such as “reduce”, “risk”, and “association” that describe the relation between plants and diseases. On the contrary, in Fig 1(b), 1(d) and 1(f), there is no explicit word to describe the relation between the plant and disease. Fig 1(g) is a case where there is no relation between a plant and disease.

We defined the annotation of relation *R*(*e*1, *e*2), where *e*1 and *e*2 represent a plant mention and disease mention, respectively, and are detected in one sentence. Relation *R* can be categorized into the presence (positive) and absence (negative) of a relation. The presence of a relation includes a therapeutic effect (ToD), an inducing effect (CoD), and association. To annotate the above relations, the locations and identifiers (IDs) of entities were annotated first. If plant and disease entities were present in the same sentence, CoD, ToD, association, or a negative relation was annotated. Corpus annotation was performed by two independent annotators.

### Guidelines for construction of a corpus of plant and disease relations

#### Entity annotation

We annotated two entity types: plants and diseases. Fig 1 shows examples of plant and disease annotation. In the annotation of disease mentions and IDs, we devised the following guidelines based on the guidelines of the NCBI disease corpus [12]. (1) Annotate the most specific disease mentions and select the best-matching merged disease vocabulary (MEDIC) IDs [13]. MEDIC [13] is the disease dictionary that reconstructs gene-related disorders in other databases: the disease branch of the medical subject headings (MeSH) [14] and Online Mendelian Inheritance in the Man (OMIM) [15] database. (2) Entities are annotated over the maximum span of text. For example, “cutaneous squamous cell carcinoma” is annotated rather than “carcinoma”. (3) Annotate if the name of the disease appears in the cell line. For example, “breast cancer (D001943)” is annotated from “MCF-7 human breast cancer cell”. (4) Do not include species names as part of a disease. Species names such as “human” are generally excluded from the preferred mention unless they are a critical part of a disease name. (5) Do not annotate general terms such as disease, syndrome, deficiency, and complications. Nevertheless, terms such as pain, cancer, and tumor should be retained. (6) Do not annotate a disease occurring in plants such as “tobacco mosaic virus.” (7) Do not annotate if the prefix is “anti-,” for example, “Anti-cancer” and “anti-inflammatory.” (8) When two different disease names appear in a single noun phrase, they represent different diseases. For example, “ovarian and breast cancer” annotates a mention of both “ovarian and breast cancer” and “breast cancer”. Annotate the ID with “ovarian cancer (D010051)” and “breast cancer (D001943)”, respectively. Nonetheless, annotate carefully if you have one disease name such as “head and neck neoplasms (D006258)”. (9) Disease-induced symptom expressions such as “diabetes-induced cardiomyopathy” are annotated for both diseases (diabetes) and symptoms (cardiomyopathy).

In case of plants, we annotated only the plant name. (1) Annotate only the name of the plant and select the best-matching merged disease vocabulary (taxonomy database) ID [16]. (2) An abbreviation (e.g., for a specific part or extract), including the plant name, is explicitly annotated with the plant. For example, in case of “H. sabdariffa aqueous extracts (HSE)” annotate “H. sabdariffa” and “HSE”. (3) Do not annotate words that represent parts of plants such as roots, stems, and leaves. (4) Do not annotate methods of processing plants such as extraction and cooking. (5) Do not annotate a plant-based product such as “chocolate” made from cocoa, and “cigarette” made from tobacco. (6) Do not annotate substances derived from plants. For example, do not annotate “caffeine”, “rg3”, and “lycopene” as a plant. (7) Do not annotate if there is an explicit statement that you will not use a plant, for example, the prefix “non-”.

#### Entity equivalence

Equivalence relations are symmetric relations between entities of the same type (plant–plant and disease–disease). Abbreviations should be annotated separately. For instance: In Fig 1(h), “AD” and “Alzheimer’s disease” can be annotated with an equivalent relation because “AD” is an acronym for “Alzheimer’s disease”.

#### Relation annotation

The relation annotation first distinguishes positive and negative relations within plant–disease pairs. Positive relations are classified as ToD, CoD, and association depending on the effect of the plant on the disease. A ToD relation represents “treatment” and “mitigation” effects of the plant on the disease. On the contrary, a CoD relation represents “occurrence” and “exacerbation” effects of the plant on the disease. An association relation occurs when there is a relation between a plant and disease, but the sentence alone cannot reveal a ToD or CoD relation.

If there is a particular trigger word describing a relation between a plant and disease in a sentence (for example, Fig 1(a), 1(c) and 1(e)), the trigger word needs to be entered into the annotation. Trigger words are verbs that explain ToD, CoD, or association relations in a sentence, for example, “induce,” “cause,” “reduce,” and “treat.” For example, “peppermint oil reduced headache” contains a ToD relation between the plant term “peppermint” and the disease term “headache” explained by the trigger term “reduced.” Another example “tobacco induces a tumor” contains a CoD relation between the plant term “tobacco” and the disease term “tumor” represented by the trigger term “induce.” In the sentence “Garlic is associated with a protective effect against stomach cancers,” “Garlic” is the plant entity, “stomach cancer” is the disease entity, and “protective effect” is the trigger term. In this case, although “effect” is a neutral explanation, this sentence contains a ToD relation due to the adjective “protective.”

Negative relations include the following categories: (1) Although a plant and disease co-occur at the sentence level, there is no description of the relation between them. (2) A sentence describing research objectives and hypotheses about plant and disease relation is considered a negative relation, as long as a result is not shown in the sentence. (3) Experimental and analysis results indicate that there is no correlation between the plant and disease. (4) Although the title contains plant and disease names, positive relations between them are not described in the title.

One sentence may contain multiple relations. For example, the sentence “These findings do not support a protective effect of (i) coffee consumption against—on total (ii) gallbladder disease, although (iii) coffee may decrease the risk of symptomatic (iv) gallstones in women.” (PMID: 11117612) contains four relation pairs ((i)-(ii), (i)-(iv), (iii)-(ii), and (iii)-(iv)). (i)-(ii) is a negative relation because “These findings do not support a protective effect” indicates category (3) of negative relations. (i)-(iv) and (iii)-(ii) are examples of category (1) of negative relations. (iii)-(iv) is a ToD category because the sentence indicates “decrease the risk.”

### Selection of abstracts for corpus construction

**Fig 2:**
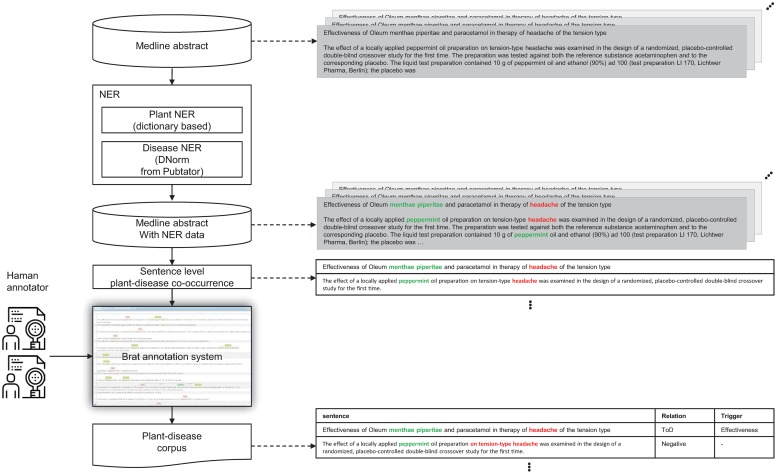
Annotation strategy.

A procedure for selecting abstracts as corpus candidates is presented in Fig 2. Medline abstracts were selected if they contained plant and disease entities in the same sentence. Plant and disease mentions were automatically annotated by named entity recognition (NER) methods. Disease mentions predicted by DNorm [17] were downloaded from PubTator [18]. DNorm uses vocabularies in MEDIC [19]. DNorm showed the best performance in the 2013 ShARe/CLEF shared task on disease normalization in clinical notes. Because there is no specialized NER tool for plants, we predicted plant mentions by dictionary-based matching using LingPipe [20].

### An annotation tool and representation

**Fig 3:**
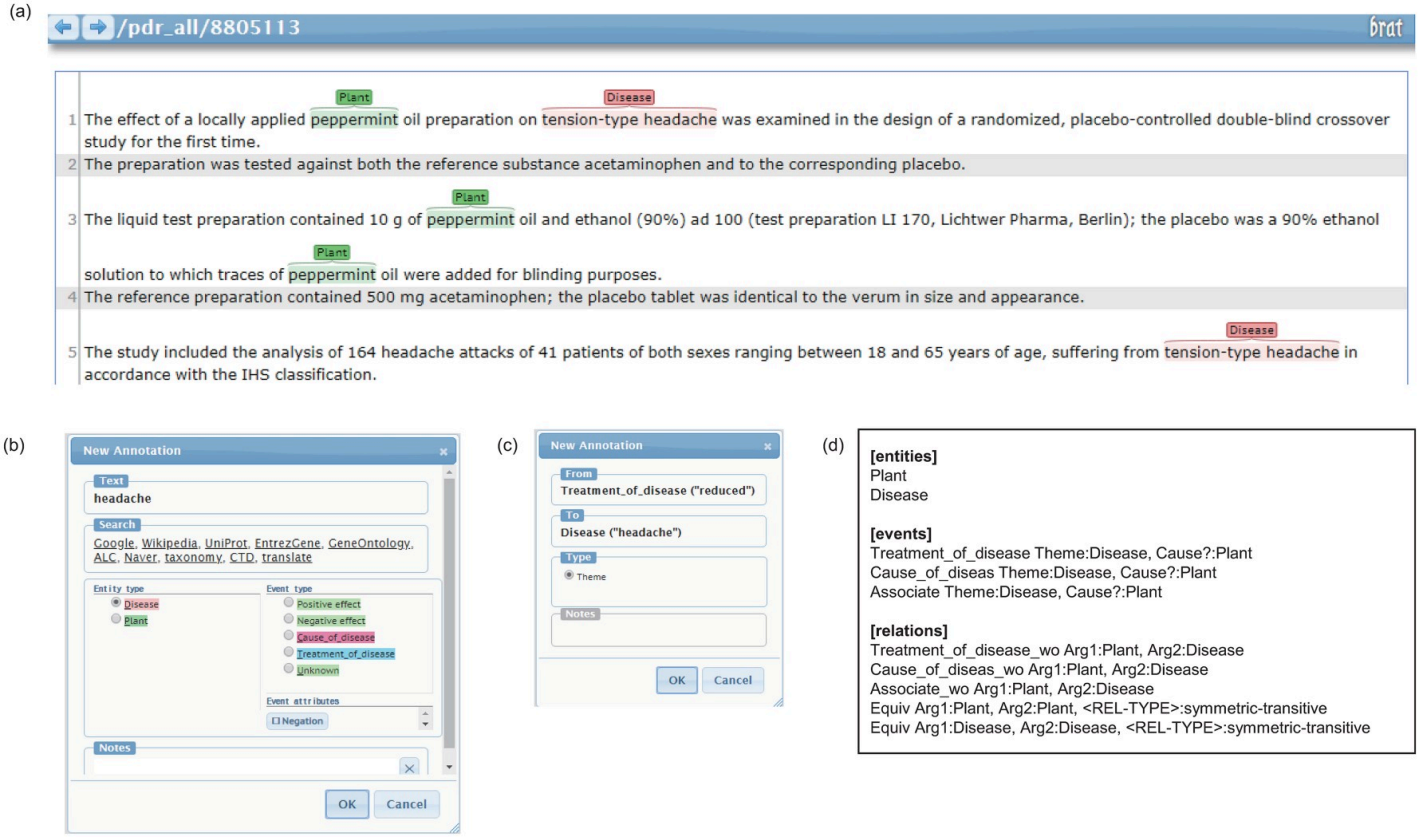
Annotation tool. (a) Annotation environment. (b) Entity annotation window. (c) Edge annotation window. (d) Definition of annotation rules in Brat.

At the annotation step, the Brat rapid annotation system [21] was employed to improve the efficiency of annotation. The Brat system is a web-based tool that visualizes annotation systems. The Brat web annotation system modified for our corpus is illustrated in Fig 3. In Fig 3(d), the entity and relation schema are designed based on the annotation guidelines.

**Fig 4:**
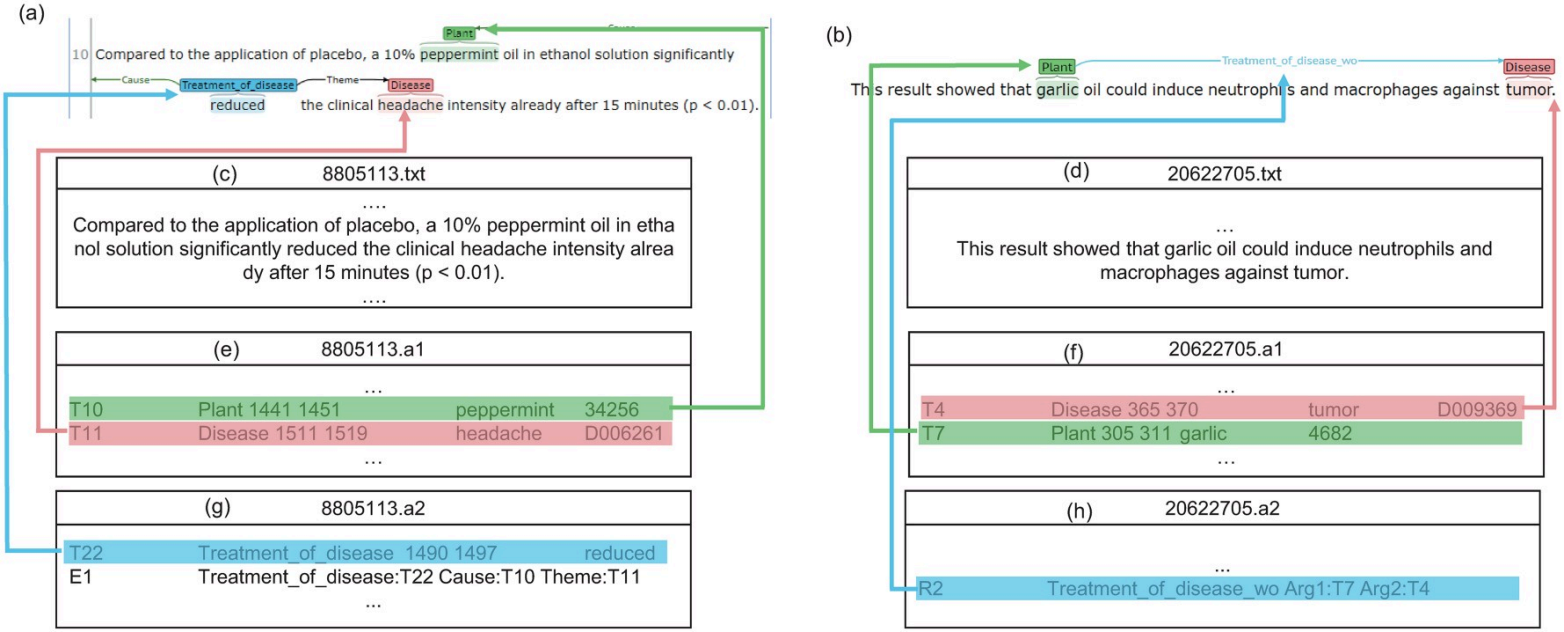
Corpus representation. (a) and (b) Corpus visualization. (c) and (d) Plain text. (e) and (f) Entity representation. (g) Representation for a relation with a trigger. (h) Representation for a relation without a trigger.

Our corpus is provided in the BioNLP shared task format [22], which is widely used in biological natural language processing. Fig 4 shows a corpus representation format. In this representation, entity mentions are indicated with the corresponding entity types, and a relation is an association of the participants in one sentence. Relations with triggers are marked among the relations between a causative plant and the resulting disease. On the contrary, relations without triggers are represented by the type of relation, the causative plant, and the target disease. The representation format consists of plain text (PubMed ID: PMID), an a1 file containing the entity information, and an a2 file containing the relation information. Entity information, in Fig 4(e) and 4(f), includes entity IDs, entity type (i.e., plant or disease), start–end offsets, entity mentions, and concept ID (i.e., NCBI taxonomy ID for a plant, and MEDIC ID for a disease). A relation with trigger information in Fig 4(g) consists of a trigger and relation. Trigger information includes a trigger ID, a relation type (CoD, ToD, or association), start–end offsets, and trigger mentions. Relation information includes relation IDs, relation type, relation trigger IDs, and cause/theme entity IDs. A relation without trigger information in Fig 4(h) includes a relation ID, relation type, Arg1 ID for a plant, and Arg2 ID for a disease. Equivalent-relation information includes relation IDs, relation type (equivalent), and two entity IDs.

### Inter-annotator agreement rates (IAAs)

Two annotators with expertise in biomedical text-mining annotated the corpus of plant and disease relations. The main annotator devised the annotation guidelines, and the main and second annotator performed annotation based on these guidelines. The annotators were allowed to use public resources such as Wikipedia and the NCBI taxonomy database.

After the two annotators performed annotation, IAAs were calculated to evaluate the quality of the annotations. A simple index, Cohen’s kappa, and a G-index [23, 24] were used. The simple index was calculated from the proportion of agreement between the two annotators. Cohen’s kappa index was employed to annotate mistakes and the coincidence between the two annotators. The G-index serves to revise the number of annotation types [25]. The IAA simple index was calculated as follows:

$$\begin{array}{c} Simple\;index\left(P_{0}\right)=\frac{number\;of\;agreed\;annotation\;unit}{N}, \end{array}$$ (1)

where *N* is the total number of annotation units. Cohen’s kappa index (*κ*) and G-index are calculated as follows:

$$\begin{array}{c} \kappa =1-\frac{1-P_{0}}{1-P_{e}}, \end{array}$$ (2)

$$\begin{array}{c} G-index=1-\frac{1-P_{0}}{1-P_{k}}, \end{array}$$ (3)

where *P*_0_ is a simple index, and *P*_*e*_ is the hypothetical probability of agreement by chance.

$$\begin{array}{c} P_{e}=\frac{1}{N^{2}}\sum_{k}n_{k1}n_{k2}, \end{array}$$ (4)

$$\begin{array}{c} P_{k}=\frac{1}{k}, \end{array}$$ (5)

where *k* is the number of categories and *n*_*ki*_ is the number of times the annotator *i* annotates category *k*. Particularly, in the calculation of IAAs for entity and trigger IAAs, we used a simple index for both “strict matches” for full-word matches and “soft matches” for partial matches.

### Plant–disease relation prediction

#### Deep neural network model

We developed a method for predicting the relations between plants and diseases to evaluate the utility of the plant disease corpus. Because relation extraction can be converted into a classification problem, various statistical machine learning methods have been successfully applied to the relation extraction task. Recently, a convolutional neural network (CNN) was applied to the relation classification task from a benchmark dataset of SemEval-2010 Task 8 [26], and a remarkable performance was achieved. This method has the potential to automatically represent features without direct effort on feature engineering. Zeng et al. [27] presented the CNN model, which combines lexical features with location features to classify relations for SemEval-2010 Task 8, surpassing the previous best-performing support vector machine (SVM) classifiers. A recurrent neural network (RNN) serves as another widely exploited model that is competitive in relation classification tasks. Xu et al. [28] proposed the use of a variant of the RNN, i.e., a long short-term memory (LSTM) network, to identify relations. They employed the LSTM network to pick up semantic information in the shortest dependency paths (SDPs).

**Fig 5:**
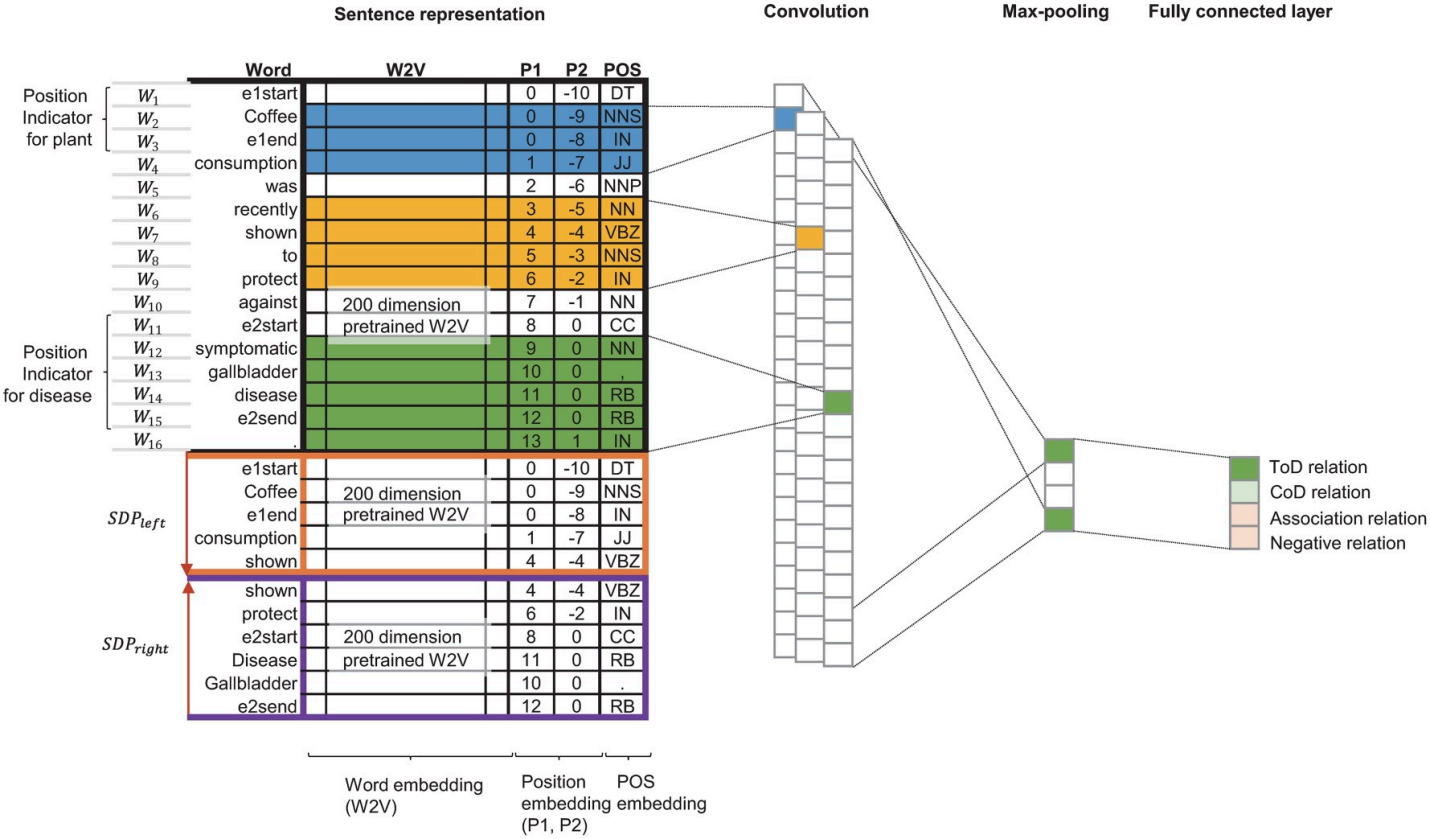
SDP-CNN architecture for plant–disease relation extraction.

Compared to the RNN, which learns through long word sequences, CNN consistently extracts local features due to its elegant properties that capture the most useful features in a flat structure and effectively abstract them. In most cases, relations are largely reflected in local words rather than in the global word order. In addition, the popularity of SDP in relation extraction tasks indicates that local information in the dependency context is useful for identifying relations. Therefore, we propose a CNN-based model to derive a more robust relation expression based on both the sentence and SDP for the plant–disease relation extraction. The model architecture is a variant of the CNN architecture described by Kim [29]. Fig 5 presents the architecture of our SDP-CNN model for the prediction of plant–disease relations. It primarily consists of the following five components: sentence representation, convolution layer, max-pooling, dropout, and softmax. A convolution layer contains varying filter windows to generate new features from sentence vectors. The sizes of filter windows in our model were 3, 4, and 5. In the max-pooling layer, the highest value over each feature map generated in the convolution layer was chosen. These features were transferred to the fully connected layer with dropout and a softmax function. The output value is a probability distribution over the classification label. In our model, the dropout rate and the number of labels were 0.5 and 4, respectively. The details about SDP are explained in the following section.

#### Shortest dependency path

**Fig 6:**
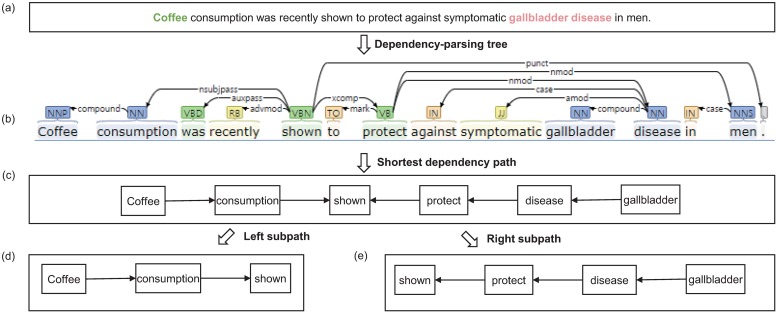
Shortest dependency parse tree. (a) An original sentence. (b) A dependency path tree for the original sentence. (c) The shortest dependency path tree for the original sentence. (d) The left subpath. (e) The right subpath.

Fig 6 shows an example of SDP in the sentence “e1start Coffee e1end consumption was recently shown to protect against symptomatic e2start gallbladder disease e2send”. *SDP*_*left*_ and *SDP*_*right*_ are constructed as follows.

$$\begin{array}{cc} SDP_{left} & :(Coffee)e1\to consumption\to protect\\ SDP_{right} & :Protect\leftarrow against\leftarrow symptomatic\leftarrow (gallbladderdisease)e2 \end{array}$$

Dependency-parsing trees are suitable for classifying relations because they focus on the behavior and agents in sentences [28]. The subpaths separated by the common ancestor nodes of the two entities provide a strong hint about the orientation of the relation. The two entities in “Coffee” and “gallbladder disease” have a common ancestor node, “shown”, that separates the SDP into two parts.

#### Sentence representation

Our SDP-CNN model first converts each token in the input sequences (sentences or paths) into a word-embedding vector and then extracts contextual features from sentences and dependency features from dependency paths. We used a pretrained word2vector (W2V) with the Medline document and Wikipedia data to vectorize words [30]. In addition, position indicators, position embeddings, and part-of-speech (POS) tags were utilized. Position indicator tags “e1start” and “e1end” were added to the front and back of the entity mentions, respectively. Position embedding (PE), *P*1 (plant) and *P*2 (disease), represent positions of words relative to the positions of a plant and disease, respectively. We employed POS tags to express the grammatical meaning of words in the word representation. The overall input sentence representation can be described as follows:

$$\begin{array}{cc} S & =[W,SDP_{left},SDP_{right}],\\ W & =[W_{1},...,W_{i}],\\ W_{k} & =[W_{k}^{W2V},W_{k}^{P1},W_{k}^{P2},W_{k}^{POS}],\\ SDP_{left} & =[W_{1}^{L},...,W_{l}^{L}],\\ SDP_{right} & =[W_{1}^{R},...,W_{r}^{R}], \end{array}$$

where *i*, *l*, and *r* are the length of a sentence, the length of a left subpath in SDP, and the length of a right subpath in SDP, respectively. *W*_*k*_∈ **R**^*d*^ is the *d*-dimensional word vector corresponding to the *k*-th word in the sentence. For initialization, pre-trained W2V was used for $W_{k}^{W2V}$ whereas random vectors were used for $W_{k}^{P1}$, $W_{k}^{P2}$, and $W_{k}^{POS}$. The dimensions for position and POS embedding were determined experimentally as shown in S1 Fig. $W_{l}^{L}\in W$ and $W_{r}^{R}\in W$ are word embeddings corresponding to SDP. To further emphasize the plant and disease entities in a sentence, dependency features from SDP were constructed in the same way as the contextual features. SDP can better describe the relations between entities if the list of essential words is repeated to describe the relation.

### Evaluation of plant–disease relation prediction

In evaluating the plant–disease relation prediction, it is difficult to use a binary F1 score because there are four types. Therefore, we evaluated the performance by means of micro and macro averages. Precision, recall, and the F1 score for micro and macro averages were calculated as follows:

$$\begin{array}{c} precision_{macro}=\frac{1}{\left|L\right|}\sum_{l\in L}P\left(y_{l},{\hat{y}}_{l}\right), \end{array}$$ (6)

$$\begin{array}{c} recall_{macro}=\frac{1}{\left|L\right|}\sum_{l\in L}R\left(y_{l},{\hat{y}}_{l}\right), \end{array}$$ (7)

$$\begin{array}{c} F_{1_{macro}}=\frac{1}{\left|L\right|}\sum_{l\in L}F_{1}\left(y_{l},{\hat{y}}_{l}\right), \end{array}$$ (8)

$$\begin{array}{c} precision_{micro}=recall_{micro}=F1_{micro}=P\left(y,\hat{y}\right), \end{array}$$ (9)

where *y* is the set of predicted (sample, label) pairs, $\hat{y}$ denotes the set of true (sample, label) pairs, *L* is the set of labels, and *y*_*l*_ represents the subset of *y* with label *l*,

$$\begin{array}{c} P\left(y,\hat{y}\right)=\frac{\left|y\cap \hat{y}\right|}{\left|y\right|}, \end{array}$$ (10)

$$\begin{array}{c} R\left(y,\hat{y}\right)=\frac{\left|y\cap \hat{y}\right|}{\left|\hat{y}\right|},and \end{array}$$ (11)

$$\begin{array}{c} F_{1}\left(y,\hat{y}\right)=\frac{2\times P\left(y,\hat{y}\right)\times R\left(y,\hat{y}\right)}{P\left(y,\hat{y}\right)+R\left(y,\hat{y}\right)}. \end{array}$$ (12)

## Results and discussion

In this section, we describe how to select Medline abstracts for corpus construction, and present the annotation qualities and statistics of the constructed corpus. Then, performances of the proposed SDP-CNN model were measured using the constructed corpus and randomly selected Medline abstracts, followed by an analysis of the distribution of plant–disease relations on the Medline scale.

### Preparation of Medline abstracts for corpus construction

We downloaded the entire Medline abstracts to a local server from PubTator data with disease mentions to select candidate sentences; the total number of abstracts was 13,408,586. In PubTator, DNorm is used for the disease NER. Disease terms appeared in 1,526,574 abstracts. PubTator also tagged species via SR4GN [31]. Nevertheless, SR4GN offered insufficient recall for plant species. Therefore, we chose a dictionary-based plant NER to find plant entities. We used the Taxonomy Database, which is classified and named for organisms as the plant dictionary. In NCBI taxonomy, a classification corresponding to the plant was chosen, and a total of 151,250 concepts and 315,173 terms were obtained. To reduce the false positives caused by synonyms, words such as anemia (ID: 12939), lens (ID: 3863), laser (ID: 62990), NAME (ID: 55581), and thymus (ID: 49990) were excluded from the plant dictionary. As a result, 823,745 abstracts contained plant mentions. Sentence level co-occurrence between diseases and plants appeared in 704,372 sentences from 469,567 abstracts, where candidate abstracts were selected randomly. Thus, a total of 200 final candidate abstracts were chosen after manual filtering of abstracts containing incorrect NER results.

### IAAs and disagreement

The two annotators annotated the entities, trigger words, and relation types. For entities, the agreement between the two annotators was measured via the simple index. The IAAs for entities were 97.329% and 98.812% for plants and diseases, respectively. For trigger words, the simple index was calculated using soft matches and strict matches, resulting in 92.183% and 78.952% for soft and strict matches, respectively.

Table 1 presents a confusion matrix of relation annotation by the two annotators. The accuracy according to the simple index, Cohen’s kappa, and G-index was 91.67%, 86.88%, and 88.89%, respectively. After the disagreements on NER annotations were resolved, 49 relations were added.

**Table 1:** IAAs for plant–disease relations.

**IAAs**		**Annotator 1**				
		**ToD**	**CoD**	**Association**	**Negative**	**Total**
**Annotator 2**	**ToD**	469	1	5	21	**96**
	**CoD**	1	155	2	11	**169**
	**Association**	3	7	23	12	**46**
	**Negative**	19	20	3	509	**549**
	**Total**	**492**	**183**	**34**	**511**	**1,260**

### Corpus statistics

Table 2 shows the corpus statistics constructed by the two annotators. From all sentences in the 200 candidate abstracts, we annotated 1,405 plant mentions with a total of 105 IDs and 1,755 disease mentions with 237 IDs. When we selected sentences with at least one plant mention and at least one disease mention, 878 plant and 1,077 disease mentions were found in 199 abstracts. The 199 abstracts contained at least one ToD, CoD, association, or negative relation, forming a total of 1,309 relations.

**Table 2:** The overall statistics for the plant–disease relation corpus.

Abstracts	Plant		Disease		Plant–disease relation
	Mention	ID	Mention	ID	
199	1,405	105	1,755	237	1,309

The numbers of relation types are given in Table 3. In summary, 725 positive relations (ToD, CoD, and association) and 584 negative relations were constructed. The total number of relations in the ToD category was 508, and the numbers of relations with or without a trigger were 432 and 76, respectively. The total number of CoD relations was 183, and the numbers of relations with or without a trigger were 157 and 26, respectively. The total number of association relations was 34, and the numbers of relations with a trigger and without one were 32 and 2, respectively. The average number of relations per abstract was 6.58.

**Table 3:** The relation statistics for the plant–disease relation corpus.

	Positive relations				Negative relations
	ToD	CoD	Association	Sum of positive relations	
**Relations with a Trigger**	432	157	32	621	584
**Relations without a Trigger**	76	26	2	104	
**Total**	**508**	**183**	**34**	**725**	

Table 4 shows the relation statistics for titles and abstracts. The titles contained 145 relations out of 202 sentences (71.78%), and the abstracts contained 1,163 relations out of 1,950 sentences (59.64%). In addition, the negative relation case in the title often refers to assumptions or experimental settings about the relation between plants and diseases. Therefore, the title contains more information about a plant–disease relation than the abstract.

**Table 4:** Statistics on relations in titles and abstracts.

	Sentences	Relations	ToD relations (%)	CoD relations (%)	Association relations (%)	Negative relations (%)
**Title**	202	145	69(47.59)	14(9.66)	1(0.69)	61(42.07)
**Abstract**	1,950	1,163	439(37.71)	169(14.52)	33(2.84)	523(44.93)

The average numbers of plant and disease mentions were 7.06 and 8.81, respectively, after the plant and disease names were normalized by taxonomy and MEDIC, respectively. Table 5 shows most the frequently appearing plants and diseases. In the CoD category, tobacco appeared 116 times, representing 63.39% of all 183 CoD relations. In the case of coffee, it can be inferred that there are various studies on the good and bad effects because they ranked second for all relation types. As for disease mentions, cancer, diabetes, asthma, and cardiovascular disease appeared most frequently.

**Table 5:** Five most frequently appearing plants and diseases for each relation.

Top	ToD		CoD		Association		Negative	
	Plant	Relations	Plant	Relations	Plant	Relations	Plant	Relations
1	tea	61	tobacco	116	tobacco	13	tobacco	151
2	garlic	58	areca	14	coffee	10	coffee	93
3	coffee	57	wheat	9	cannabis	4	tea	61
4	ginger	22	digitalis	8	apple	1	garlic	46
5	soybean	19	coffee	5	pear	1	ginseng	21

Table 6 presents the trigger words in ToD, CoD, and association categories. Trigger terms were normalized by considering the tense and prepositions to obtain statistics. For example, “reduce” is a normalized form of “reducing” and “reduced.” The total number of triggers in the ToD category was 432. The five most frequently appearing triggers—“effect,” “reduce,” “prevent,” “protect,” and “decrease”—occurred in 63.19% of all ToD relations. The total number of trigger words for CoD relations was 157. The five most frequently appearing trigger words for CoD relations—“relate,” “associate,” “induce,” “increase,” and “risk” accounted for 77.7% of all CoD relations. These event trigger words seem to show a causal relation.

**Table 6:** Five most frequently appearing plants and diseases for each relation.

Top	ToD		CoD		Association	
	Trigger	Relations	Trigger	Relations	Trigger	Relations
1	effect	71	relate	57	associate	26
2	reduce	69	associate	23	effect	4
3	prevent	62	induce	20	relate	2
4	protect	36	increase	13	influence	1
5	decrease	35	risk	9	-	-

### Relation prediction

#### Performance of four-class-relation prediction

We assessed the performance of the proposed SDP-CNN model and compared its performance with that of other models as described in Table 7. As the baseline, we utilized the Turku Event Extraction System (TEES) [32], which showed excellent performance on the extraction of biomedical events via SVM. Other deep-learning–based models were compared. Based on Yoon Kim’s model [29], various techniques were next applied to the CNN model including position indicators, position embeddings, POS tags, and SDP. We experimented with a total of seven models, and performance was evaluated based on ten-fold cross-validation, where abstracts were divided into ten subsets.

**Table 7:** Performance of the plant–disease prediction model applying the suggested plant–disease corpus.

Data	Model	Embedding	Macro			Micro
			Recall	Precision	F1	F1
**Relations with a trigger (1,205 relations)**	SVM (event extraction)		0.612	0.598	0.605	0.622
	CNN	position indicator	0.545	0.692	0.561	0.757
	CNN	position indicator + position embedding	0.551	0.639	0.567	0.765
	CNN	position indicator + POS	0.552	0.630	0.568	0.765
	CNN	position indicator + position embedding + POS	0.545	0.671	0.565	0.763
	SDP-CNN (only SDP path*)	position indicator + position embedding + POS	0.541	0.610	0.557	0.749
	SDP-CNN	position indicator + position embedding + POS	0.557	0.647	0.578	**0.760**
**Relations with/without a trigger (1,309 relations)**	SVM (relation extraction)		0.661	0.612	0.636	0.689
	CNN	position indicator	0.554	0.614	0.565	0.749
	CNN	position indicator + position embedding	0.565	0.624	0.575	0.763
	CNN	position indicator + POS	0.543	0.829	0.562	0.756
	CNN	position indicator + position embedding + POS	0.563	0.622	0.574	0.762
	SDP-CNN (only SDP path*)	position indicator +position embedding + POS	0.535	0.548	0.539	0.733
	SDP-CNN	position indicator + position embedding + POS	0.563	0.574	0.567	**0.764**

* We excluded relations that could not find the SDP in the sentence, resulting in 1,177 and 1,263 relations for the cases of trigger and with/without trigger, respectively.

We conducted experiments in two cases: for relations with trigger words and for all relations with or without trigger words. Of all 1,307 relations, the numbers of relations with trigger and without trigger were 1,205 and 104, respectively. In a relation without a trigger, the performance of SVM was characterized by a macro F1 score of 0.605 and a micro F1 score of 0.622. The most basic CNN model with position indicators in the same data yielded a macro F1 score of 0.561 and a micro F1 score of 0.757. The SVM model showed higher performance than the CNN model for the macro score, where the accuracies of the four classes were averaged and its score heavily depends on association relation with the smallest number of instances. However, the CNN model outperformed the SVM model for the micro score, confirming that the overall accuracy was better. It was observed that TEES used many attributes extracted from a sentence, e.g., sentence structure, bag of words, and n-grams, as classification features. Nevertheless, in micro average measurement, the CNN models involving word vectors outperformed the TEES model.

Among the machine learning models, SDP-CNN was the best-performing model. The macro F1 score and micro F1 score was 0.567 and 0.764, respectively. In the SDP-CNN (only SDP) model, except for the original sentence, the macro F1 score and micro F1 score was 0.539 and 0.733, respectively. After testing SDP, we could confirm that full sentence information helps to predict a relation between a plant and disease.

Notably, when all the relation data with or without trigger words were analyzed, the accuracy rates of the models did not show significant differences compared to the analysis where only relations with a trigger were analyzed. This might be because the number of relations without a trigger was small compared to all data (8.6% of relations).

The CNN model showed lower performance than the SDP-CNN model. We assumed that the SDP reduces the distance between plant and disease, resulting in reduced long-term dependency. In the original sentence, the mean distance between the plant and disease was 9.91 and 11.06 in relations with a trigger and in relations without a trigger, respectively. However, the mean distance between the plant and disease in SDP was 5.82 and 6.25 in relations with a trigger and in relations without a trigger, respectively (S2 Fig). Therefore, the proposed SDP-CNN model might improve the prediction accuracies.

#### Performance of binary relation prediction

We evaluated the performance of the proposed model for binary classification. Experiments on the binary classes were conducted in four cases: (i) the ToD relations are positives and the rest are negatives, (ii) the CoD relations are positives and the rest are negatives, (iii) the association relations are positives and the rest are negatives, and (iv) ToD, CoD, and association relations are positives and the rest are negatives. Table 8 indicates that the F1 scores were 0.907, 0.856, 0.795, and 0.925, respectively. It was observed that the proposed method is especially more accurate in predicting a therapeutic effect (ToD) and a simple positive plant–disease relation (ToD, CoD, and association).

**Table 8:** Performance of the SDP-CNN model on prediction of plant–disease binary relations.

Positive class	Recall	Precision	F1
**Treatment of disease**	0.906	0.907	0.907
**Cause of disease**	0.851	0.862	0.856
**Association**	0.756	0.839	0.795
**Treatment of disease + Cause of disease + Association**	0.918	0.932	0.925

#### Effect of pretrained word embedding

We also evaluated the performance of pretrained W2V. Table 9 shows the performance comparison according to W2V. The experiment was evaluated by means of the best-performing model: SDP-CNN. We used 300 dimensions of Google News W2V [33] and 200 dimensions of PubMed-related W2V [30]. We compared the randomly generated vector and pretrained W2V for each dimension. We also compared non-static models that train word vectors as well as static models that do not train word vectors. The best performance was manifested by the model with non-static and W2V vectors constructed from PubMed, PubMed Central, and Wikipedia. The next best performance was shown by Google News W2V. Although Google News W2V was created with the largest amount of data, the W2V involving data from PubMed (the domain of this corpus) performed better.

**Table 9:** Performance (micro F1 score) comparison analysis according to pretrained W2V and static/non-static SDP-CNN model.

Embedding size	300		200					
W2V	Random	Google news	Random	Disease	PubMed	PMC	PMC + PubMed	Wiki + PMC + PubMed
**Static**	0.697	0.715	0.678	0.710	0.738	0.754	0.749	0.752
**Non-static**	0.691	0.725	0.670	0.711	0.749	0.757	0.759	0.764

### Medline scale analysis

We applied the proposed SDP-CNN model at the Medline scale. First, we extracted sentences that contain plants and diseases in one sentence. Medline abstracts and disease annotation were collected from PubTator [18]. The disease mentions in PubTator were predicted using DNorm [17]. The plant names were predicted with a dictionary-based NER using taxonomy database [16]. The total number of co-occurrences was 353,724, and the plant and disease relations from these co-occurrences were predicted using the SDP-CNN model.

#### Manual validation of predicted relation

We randomly extracted 483 relations from the predicted relations, in which both plant and disease NER were correct. When the main annotator manually validated the performance, a precision of 0.706 was obtained, which was similar or better than the precisions obtained from the cross-validation of the corpus as shown in Table 7.

#### Distribution of plants and diseases on the Medline scale

**Fig 7:**
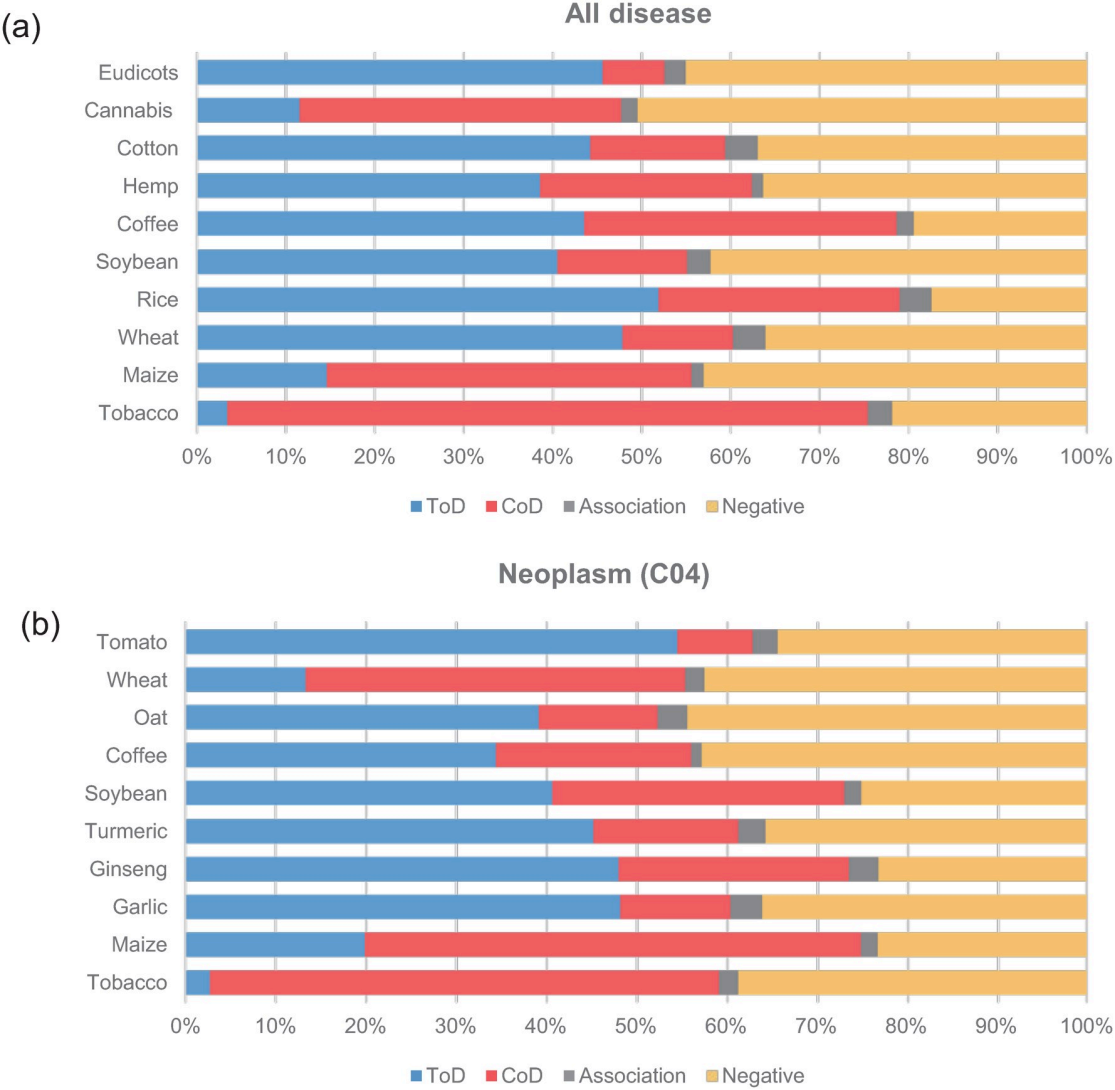
Distribution of relations according to the plant. (a) All disease (b) Neoplasms (C04).

We examined the distribution of relation types between plants and diseases on the Medline scale. The distribution of relation types for the ten most common plants across all diseases is shown in Fig 7(a). Tobacco and cannabis accounted for 11.2% and 3.0% of the total plant mentions, respectively. The distribution of the relation types of the ten most common plants in neoplasms (MeSH tree number [14]: C04.*) is shown in Fig 7(b). Tobacco mainly showed CoD relations for all diseases as well as neoplasms whereas for coffee, the proportion of ToD and CoD relations was similar.

For inflammation (D007249), the ten most common plants are shown in Table 10. In particular, turmeric, ginseng, garlic, and ginkgo were found to be high-ranking plants that are known to have anti-inflammatory properties. This Medline scale analysis shows that our proposed corpus and the relation prediction model provide useful information on the relation between plants and diseases.

**Table 10:** Top 10 plants in inflammation (D007249) in Medline scale analysis.

Common name	Taxonomy ID	# relations
**Tobacco**	4097	76
**Cotton**	3635	61
**Turmeric**	136217	21
**Coffee**	13443	14
**Ginseng**	4054	13
**Birches**	3504	12
**Rice**	4530	11
**Wheat**	4565	11
**Peanut**	3818	11
**Garlic**	4682	10
**Ginkgo**	3310	10

## Conclusion

The corpus of relations between plants and diseases constructed in this study is unique and can form the basis for research on extracting knowledge from biomedical texts. In this study, two annotators created the guidelines and the corpus for the relations between plants and diseases, resulting in a total of 1,309 relations from 199 abstracts. Although the corpus size may be small, it has high IAA scores. Thus, it can serve as a gold standard dataset for studies on plant–disease relations.

Moreover, we created the SDP-CNN model to predict plant–disease relations for evaluating the reliability of the corpus. The micro F-score was 0.764. Thus, using the constructed corpus and the proposed model, more plant–disease relations can be extracted from Medline abstracts.

## Supporting information

S1 Fig Prediction accuracy for embedding size. (A) is the F1 (micro) score for the embedding size of PE. (B) is the F1 (micro) score for the embedding size of POS.
(TIF)

S2 Fig Box plot of distance(word count) between the plant and disease. The word distance in the original sentence and the SDP are analyzed by dividing them by the presence or absence of the trigger word.
(TIF)

S1 File A relation corpus in Excel format. Provides information about PMID, sentence ID, relation ID, a sentence with entity indicator, plant mention, plant ID, disease indication, disease ID, relation category, and trigger mention in the units of relation.
(XLSX)
