# Supplementary figures and images for: A corpus of plant–disease relations in the biomedical domain
Source: PLoS One. 2019 Aug 28;14(8):e0221582. doi: 10.1371/journal.pone.0221582 (PMC6713337; doi:10.1371/journal.pone.0221582)

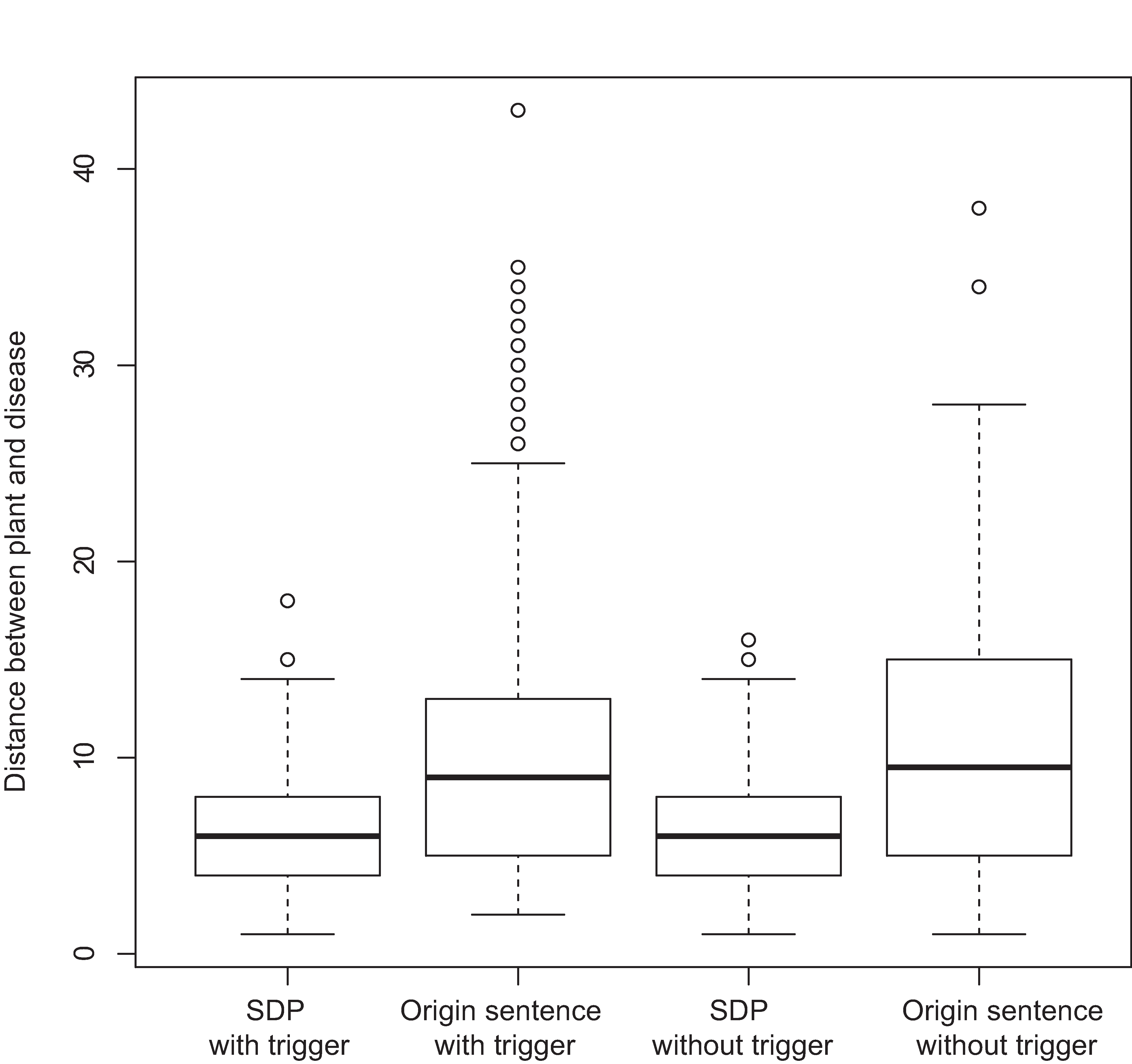

Supplement: S1 Fig — (A) is the F1 (micro) score for the embedding size of PE. (B) is the F1 (micro) score for the embedding size of POS. (TIF) [file pone.0221582.s001.tif]

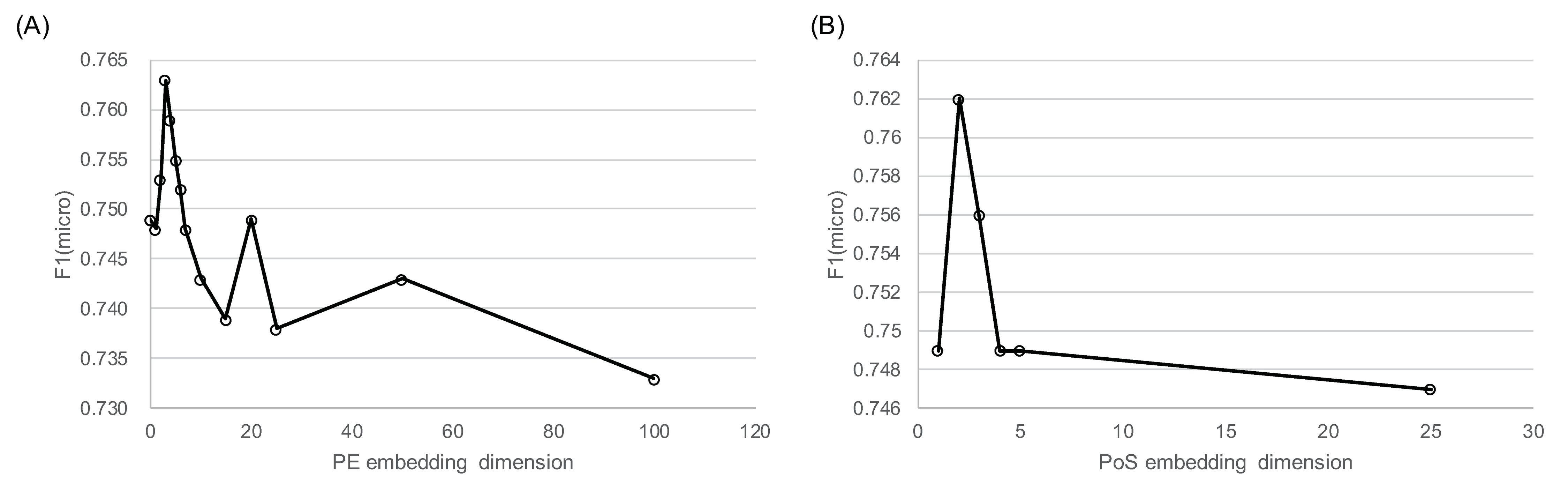

Supplement: S2 Fig — The word distance in the original sentence and the SDP are analyzed by dividing them by the presence or absence of the trigger word. (TIF) [file pone.0221582.s002.tif]
